# The COVID-19 pandemic in sub-Saharan Africa: The significance of presumed immune sufficiency

**DOI:** 10.4102/ajlm.v12i1.1964

**Published:** 2023-01-30

**Authors:** Abel O. Idowu, Yusuf O. Omosun, Joseph U. Igietseme, Anthony A. Azenabor

**Affiliations:** 1 Department of Pharmaceutical Microbiology and Biotechnology, Faculty of Pharmacy, College of Medicine, University of Lagos, Lagos, Nigeria; 2 Department of Microbiology, Biochemistry and Immunology, Morehouse School of Medicine, Atlanta, Georgia, United States; 3 Centers for Disease Control and Prevention (CDC), Atlanta, Georgia, United States

**Keywords:** COVID-19, coronavirus, immune response, sub-Saharan Africa, infectious diseases

## Abstract

A novel coronavirus known as severe acute respiratory syndrome coronavirus 2 (SARS-CoV-2) was first reported in China in 2019 and later ignited a global pandemic. Contrary to expectations, the effect of the pandemic was not as devastating to Africa and its young population compared to the rest of the world. To provide insight into the possible reasons for the presumed immune sufficiency to coronavirus disease 2019 (COVID-19) in Africa, this review critically examines literature published from 2020 onwards on the dynamics of COVID-19 infection and immunity and how other prevalent infectious diseases in Africa might have influenced the outcome of COVID-19. Studies characterising the immune response in patients with COVID-19 show that the correlates of protection in infected individuals are T-cell responses against the SARS-CoV-2 spike protein and neutralising titres of immunoglobin G and immunoglobin A antibodies. In some other studies, substantial pre-existing T-cell reactivity to SARS-CoV-2 was detected in many people from diverse geographical locations without a history of exposure. Certain studies also suggest that innate immune memory, which offers protection against reinfection with the same or another pathogen, might influence the severity of COVID-19. In addition, an initial analysis of epidemiological data showed that COVID‑19 cases were not severe in some countries that implemented universal Bacillus Calmette–Guerin (BCG) vaccination policies, thus supporting the potential of BCG vaccination to boost innate immunity. The high burden of infectious diseases and the extensive vaccination campaigns previously conducted in Africa could have induced specific and non-specific protective immunity to infectious pathogens in Africans.

## Introduction

In December 2019, a group of pneumonia cases of undetermined origin where patients presented with clinical signs that resemble viral pneumonia was first described in the city of Wuhan, Hubei, China.^1^ Analysis of samples from the lower respiratory tract by deep sequencing implicated a novel coronavirus that was named 2019-nCoV.^1,2^ On 11 March 2020, the World Health Organization (WHO) declared the coronavirus disease 2019 (COVID-19), caused by severe acute respiratory syndrome coronavirus 2 (SARS-CoV-2), a worldwide pandemic. As of 07 September 2021, SARS-CoV-2 had cumulatively infected over 224 686 497 people and caused 4 566 167 deaths in 225 countries, with a case fatality rate of 2.1%.^3^ The spectrum of COVID-19 manifestation may vary from a self-limiting mild respiratory tract infection to a severe pneumonia that could lead to the failure of multiple organs and death.^4^ The common symptoms, including fever, cough, and difficulty in breathing, typically emerge within 2–14 days after exposure.^4,5^ Certain individuals may, however, exhibit minimal or no symptoms even with a positive reverse transcription polymerase chain reaction test.^6,7,8,9^ The risk of death is greater among older or immunocompromised patients,^10^ and the ability of asymptomatic individuals to efficiently transmit the virus^11^ has made it difficult to control the epidemic.^6^

Coronaviruses are characteristically single-stranded, positive-sense RNA viruses that disseminate widely in birds, humans, and other mammals, and can cause enteric, respiratory, neurological, and hepatic diseases.^12,13^ Six species of coronavirus species cause human diseases, and four of them, NL63, HKU1, hCoV-229E, and OC43, are predominantly responsible for mild respiratory infections.^14^ The first SARS-CoV, which emerged in 2002,^15,16,17^ and Middle East respiratory syndrome coronavirus, which became known in 2012,^18^ are two deadly novel coronaviruses that cause severe pneumonia and have appeared occasionally in different areas. However, in contrast to SARS-CoV-2, the number of confirmed SARS-CoV and Middle East respiratory syndrome coronavirus cases are smaller (8100 and 2500) because of limited transmission from person to person. The probability of the periodic emergence of novel coronaviruses is high as a result of its extensive dissemination among humans and animal species, high genetic variability and recurrent genomic recombination, in addition to other factors such as growing human and animal interactions, regular cross-species infections, and rare spillover events.^19,20^ The sequencing of the 2019-nCoV genome showed that it is phylogenetically similar to particular beta-coronaviruses found in bats that belong to the sarbecovirus subgenus in the Coronaviridae family.^2^

**FIGURE 1:**
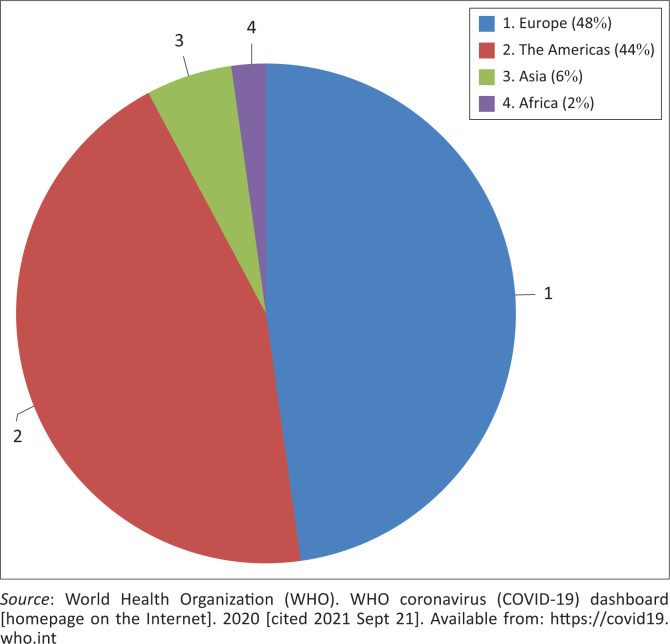
Global distribution of COVID-19 cases per million people as of 07 September 2021.

Contrary to expectations that the African region would quickly succumb to the devastation of the rampaging COVID-19 pandemic, fewer cases, as shown by the number of cases per million people, are being reported in Africa compared to other parts of the world (Figure 1^3^). Besides, a comparison of the incidence of cases and the case fatality ratio among some selected African and Western countries (Table 1^3^) showed that the case fatality ratio and number of cases in Africa were relatively low. This suggests that Africa was mildly impacted by the pandemic despite the weak health infrastructure. This review summarises current literature on the dynamics of infection and immunity to SARS-CoV-2 and other infective diseases in Africa and the unique characteristics of the sub-Saharan African population that might have impacted the course and severity of the pandemic. We also describe the host immune response mechanisms to other microbial infections and the possible reasons for a presumed immune sufficiency to COVID-19 in the African population.

**TABLE 1:** Cumulative total number of COVID-19 cases, deaths and case fatality ratio reported in some Western and African countries between 05 February 2020 and 07 September 2021.

Country	Number of cases	Number of deaths	Case fatality ratio
United States	40 700 160	662 637	1.62
United Kingdom	7 377 676	135 336	1.80
France	6 692 706	113 461	1.70
Italy	4 601 749	129 885	2.80
Spain	4 938 919	87 676	1.80
Germany	4 084 721	94 458	2.30
Nigeria	198 786	2 590	1.30
Ethiopia	322 737	4 907	1.50
Kenya	243 456	4 902	2.00
South Africa	2 854 234	84 751	2.97
Zambia	207 836	3 629	1.70
Botswana	165 644	2 337	1.40

*Source*: World Health Organization (WHO). WHO coronavirus (COVID-19) dashboard [homepage on the Internet]. 2020 [cited 2021 Sept 21]. Available from: https://covid19.who.int

The terms ‘epidemiology and immunity to COVID-19’, ‘COVID-19 and associated coronaviruses’, ‘prevalent infectious diseases in Africa’, ‘host immune response mechanisms to viral and other microbial infections’, and ‘immune sufficiency to COVID-19 in the African population’ were used to carry out a systematic search on relevant search engines and websites such as Google, Google Scholar, PubMed, African Journals Online, World Health Organization, Center for Disease Control and Prevention, Nigeria Centre for Disease Control, and Africa Centre for Disease Control and Prevention. We included all relevant studies published in the English language between 2000 and 2021.

## Immunity to COVID-19

The specific aspects of immunity that define both the protective and pathogenic reactions to SARS-CoV-2 infection are not clear. Though some components of the immune response to SARS-CoV-2 infection appear to be unique, some aspects are similar to the mechanisms of immunity to other human and animal infections. An understanding of the protective components of the immune reaction to SARS-CoV-2 infections is important for effective vaccine development. As is the case with many infectious diseases, an immune reaction to SARS-CoV-2 involves both cell-mediated and antibody-facilitated responses.^21^ T-cell responses against the viral spike protein have been shown to correlate well with the neutralising titres of immunoglobin G and immunoglobin A antibodies.^22,23,24^ This observation could have important implications for vaccine design and the development of durable immunity. As the primary inflammatory cells, the CD8+ T-cells play a crucial function in clearing the virus. The level of inflammation during SARS-CoV-2 infection is significantly reliant on the association between the CD4+/CD8+ ratio and the total lymphocytes, namely CD4+ T-cells, CD8+ T-cells, and B-cells and natural killer cells.^10^ In both mild and severe cases, there is a decrease in the absolute numbers of T lymphocytes, CD4+ T-cells, and CD8+ T-cells, but the decrease is more in severe than in mild cases.^10^ The implication of the lymphopenia observed is that it reduces the ability of the immune system to fight the infection and increases the chances of severe illness. Multivariate data analysis showed that a reduction in B-cells and CD8+ T-cells, and a rise in the CD4+/CD8+ ratio can independently predict a poor treatment outcome.^10^ In the context of the African population where immune sufficiency is presumed, it is expected that a reduction in the numbers of T lymphocytes would be limited but this needs to be investigated. Interferon-γ expression by CD4+ T-cell induction also tends to reduce in severe cases compared to moderate cases.^4^ It takes between 10 and 21 days after infection for antibody response to develop in most SARS-CoV-2-infected persons, and it takes between 6 and 15 days after the onset of disease for the immunoglobin M and immunoglobin G antibodies to SARS-CoV-2 to develop.^25,26,27,28,29^ In mild cases, antibody development can take up to 4 weeks or longer and may even be undetectable in a small number of cases. The basis of protective immunity from a successive infection is the immune memory derived from either primary infection or immunisation.^30,31,32^ Recently, a predominantly cross-sectional study, which also included a longitudinal component of 188 recovered COVID-19 cases, assessed the involvement of the CD4+ T-cell, CD8+ T-cell, and humoral components of adaptive immunity in the immune memory. The result showed that COVID-19 infection is followed by the generation of considerable immune memory involving all types of adaptive immune components,^21^ thus suggesting that activation of antibodies or T-cell reactivity are correlates of protection against COVID-19 infection. The question is: how long does the immune protection last?

In other coronaviruses, antibody levels decline between 12 and 52 weeks from the inception of symptoms, resulting in homologous reinfections.^33^ In contrast to SARS-CoV-1 infections where immunoglobin G antibody levels could be sustained for two years in 90% of patients and for three years in 50% of patients,^34^ immunoglobin M and immunoglobulin G antibody levels in SARS-CoV-2 could only be sustained for over seven weeks^35^ or until day 49 in at least 80% of the cases.^36^ A recent study showed that 95% of study subjects retained immune memory lasting approximately six months after infection.^21^

## Disease dynamics of COVID-19 in sub-Saharan African countries

**FIGURE 2:**
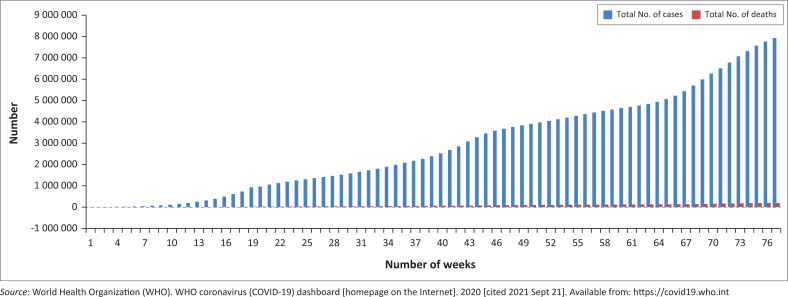
Cumulative total number of COVID-19 cases and deaths reported in Africa between 05 February 2020 and 07 September 2021.

The incidence of SARS-CoV-2 infections in sub-Saharan Africa was initially low but increased rapidly over time until it became widespread in virtually all countries. As of 07 September 2021, the total number of COVID-19 cases reported in 55 African countries was 7 926 999, with 200 045 deaths and a case fatality ratio of 2.5%.^37^ This corresponds to 3.6% of the total number of cases and 4.4% of the total number of deaths reported globally. Within Africa, the total number of COVID-19 cases and deaths have varied between countries and with time^37^ (Figure 2^3^). As of 07 September 2021, South Africa, which is the epicentre of the pandemic in Africa, had recorded the highest incidence of validated COVID-19 infections (2 819 945) in sub-Saharan Africa, followed by Ethiopia (314 984), Kenya (240 172), Zambia (207 114), Nigeria (195 511), and Botswana (162 186).^37^ Although Nigeria is the most populous African nation, it has the fifth largest case count in sub-Saharan Africa and the largest outbreak in West Africa.^37^ The number of cases being reported in Africa compared to the other regions of the globe may have been partly influenced by the low level of testing,^37^ suggesting a high likelihood of many undetected cases. The level of testing in Africa has been hindered by factors that include limited laboratory capacity, availability and accessibility of testing sites, cost of testing, low health literacy, and the stigma associated with testing positive.^38^ Also, certain characteristics peculiar to Africa in terms of age, health, lifestyle, and previous experiences with other infectious disease outbreaks might have impacted how the pandemic is playing out in Africa.

With a median age of under 20 years, Africa has a relatively young population compared to European Union countries (43 years) and China and the United States (38 years).^39^ Besides, only 3% of the African population is above 65 years, which is the threshold for significant chances of complications and deaths due to COVID-19.^40^ In Nigeria, the most populous country in Africa, over 44% of the population is below the age of 15 years, the age group with the fewest number of reported cases and mortality.^41^ However, there are fears that other demographic features peculiar to Africa such as frequent migration across borders, a high number of displaced persons, rapid urbanisation, and large households^41^ could complicate prevention and mitigation efforts, thereby driving up the case count and the number of deaths. In addition, factors like the worsening burden of existing health conditions such as tuberculosis and HIV/AIDS^42,43^ with their attendant effects on the population’s immune status, as well as the high incidence of non-communicable diseases such as hypertension and heart diseases, increase the chances of complications from COVID-19 infections.

## Infectious diseases in sub-Saharan Africa

Infectious diseases, responsible for a minimum of 69% of deaths in Africa,^44^ are a serious challenge in Africa due to various underlying factors such as poverty, poor environmental hygiene, overcrowded housing, limited access to affordable healthcare, and lack of basic health education.^45,46,47^ Malaria, HIV/AIDS, tuberculosis, Ebola disease, Lassa fever, guinea worm, elephantiasis, river blindness, lower respiratory tract infection, and diarrheal disease are prevalent or have had outbreaks in Africa.^48^ There are six infectious diseases in the 2019 WHO report of the top 10 causes of disability-adjusted life years in sub-Saharan Africa.^49^ These include malaria, AIDS, lower respiratory tract infections, diarrheal infections, meningitis and tuberculosis.^49^ The spread of these diseases could occur more easily in countries with inadequate infrastructure and fragile health systems.

Malaria, caused by plasmodium species, is a vector-borne parasitic disease that infects humans via the bites of infected female Anopheles mosquitoes.^50^ In 2020, the WHO estimated that 3.2 billion people across 96 countries run the risk of having malaria, with the highest burden being in sub-Saharan Africa. This region accounts for 94% of global cases and 94% of deaths, 67% of which are in children aged under five years.^50^ The HIV is a retrovirus that progressively destroys and weakens the immune system of an infected person until it becomes vulnerable to opportunistic infections. If untreated, the HIV infection can progress to the most complex stage of the disease referred to as AIDS, resulting in death.^51,52^ Over 37.7 million people are living with HIV globally, with 680 000 annual deaths.^53^ Sub-Saharan Africa is the worst affected, being home to 67% of people living with HIV and 39% of new HIV cases.^53^ Although most people living with HIV have higher co-morbidities and experience more severe outcomes from COVID-19 than people without HIV, most of them did not have access to COVID-19 vaccines as of mid-2021.^53^

Tuberculosis is caused by the bacterium *Mycobacterium tuberculosis* and is an infectious disease that commonly affects the lungs and is transmissible from person to person through coughing, sneezing, or spitting.^54,55^ In 2019, there were 10 million cases of tuberculosis (208 000 were in HIV-positive people) and over 1.2 million deaths due to tuberculosis (15% of all deaths were related to HIV).^56^ Africa ranked second in the global burden of new tuberculosis cases (25%) in 2019.^56^ Ebola virus causes an acute, contagious, and deadly disease that is transmitted from person to person through close contact with fluids from the body of symptomatic and asymptomatic living patients or dead victims.^57,58^ Periodic, remote outbreaks of Ebola have occurred in many Central African countries.^59,60^ The most widespread outbreak of Ebola occurred in Guinea, West Africa, in 2014 from where it spread to neighbouring Sierra Leone and Liberia, killing an estimated 11 000 people by April 2016.^61,62,63^

## Immune response to microbial infections

**FIGURE 3:**
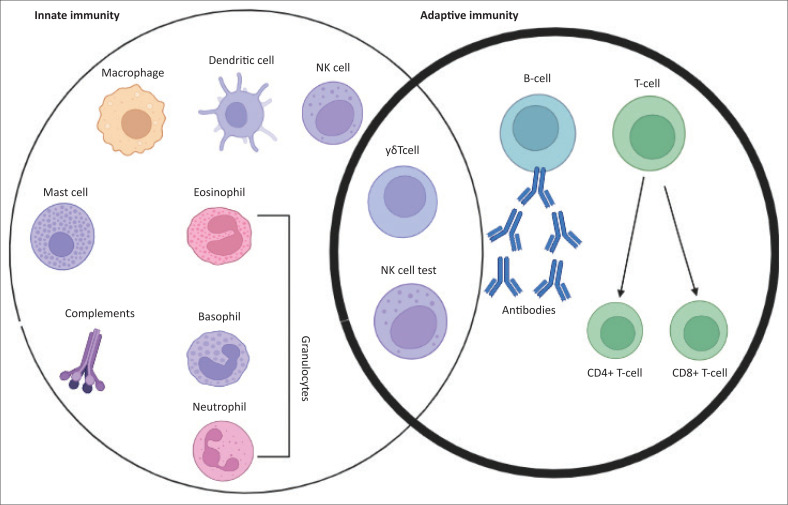
Cells involved in innate and adaptive immune responses against pathogens. The immune cells from innate immunity which provide the first line of defence are generated by myeloid lineage cells and they include monocytes, macrophages, erythrocytes, platelets, and granulocytes. Cells from the adaptive immune system are responsible for the second line of defence and consist of natural killer cells, B-cells, and T-cells that come from lymphoid progenitor cells. Recognition of external antigens by the macrophages and dendritic cells of the innate immune system triggers the naive CD8+ and CD4+ T-cells of the adaptive immune system.

The human body protects itself from microbial infections through the immune system made up of innate and adaptive arms that are not only linked but consist of an extraordinarily diverse compilation of cells (Figure 3; compiled in bioRENDER, https://biorender.com/). The innate immune system is the host’s first-line defence against invading pathogens, while adaptive immune responses are triggered by the host’s immune recognition controls.^64^ With the help of molecular patterns that are pathogen-related, the innate immune system recognises repetitive evolutionarily conserved molecules on pathogens by using germline-encoded pattern recognition receptors such as C-type lectin receptors, Toll-like receptors, nucleotide-binding oligomerisation domain-like receptors, and retinoic acid-inducible gene I-like receptors.^65^ The immune cells that mediate innate immune defences such as natural killer cells, complement proteins, and phagocytic cells like monocytes, macrophages, and neutrophils do not have effector mechanisms that induce immunological memory.^66^

Adaptive immunity comprises cell-mediated and humoral immunity, which have a wider and fine-tuned range of recognition because of exposure to variable antigens. The unique traits of the adaptive immune system are pathogen-specific immunological memory, immune effector functions, tolerance, regulation of host immune homeostasis, and increased production of inflammatory mediators.^67^ When the innate immune system is exposed to invading organisms in the form of vaccines (Bacillus Calmette–Guerin [BCG] vaccine, measles vaccine, etc.) or bacterial and fungal complex biomolecules (lipopolysaccharides, β-glucan, etc.), the immune cells undergo metabolic alteration or epigenetic reprogramming.^68,69^ These changes, described as ‘trained immunity’, elicit an elevated response when challenged afterwards by the first trigger or different unrelated organisms or molecules through T- or B-cell-independent responses, leading to increased production of inflammatory mediators.^70,71,72^ Unlike the conventional, more precise, epitope-dependent adaptive immune memory that is mediated by lymphocytes, the control of trained immunity is facilitated by a unique mechanism that is not very specific and lasts only for a short while.^73^ Nevertheless, both fulfil the same major task of eliciting a faster and more robust response that destroys the pathogens and improves the survival of the host. Trained immunity could be beneficially applied to develop improved vaccines^74,75^ and limit the adverse effects of inflammatory diseases.^76^

## Possible reasons for a presumed immune sufficiency in sub-Saharan Africa

### Adaptive immunity-mediated pre-existing immunity to SARS-CoV-2

COVID-19 infections are often asymptomatic or mildly symptomatic but may be severe, mostly among vulnerable patients, presenting as acute respiratory distress.^77^ The mild pattern of infection in many patients may be due to an exposure-related cross-immunity to other endemic common cold coronaviruses.^78^ These viruses cause mild self-limiting respiratory infections and circulate widely in the human population where globally, more than 90% are seropositive for at least three of the common cold coronaviruses.^79^ There has been evidence that supports the likelihood that circulating memory B-cells and neutralising antibodies developed to one type of coronavirus may cross-react with other members of the coronavirus family.^78^ Emerging data from studies conducted in unexposed donors from different geographical locations revealed that 20% – 50% had lymphocytes with significant reactivity to pools of SARS-CoV-2 antigen peptides.^22,23,24,80,81^ There is also speculation that the SARS-CoV-2-specific T-cells in unexposed individuals originated from the memory T-cells obtained from contact with common cold coronaviruses.^82,83^ In a study conducted in Germany, the highest T-cell reactivity occurred against a pool of SARS-CoV-2 spike peptides that are homologous to common cold coronavirus spike proteins.^24^ The occurrence of SARS-CoV-2 pre-existing primed T-cells and neutralising antibodies acquired through previous exposure to endemic coronaviruses could slow down disease transmission and limit the number of cases and mortality.^78,84^ Individuals with a high level of pre-existing CD4+ T-cells with SARS-CoV-2 recognition memory may elicit a faster and more robust immune response upon exposure, thus limiting the severity of the disease. In such individuals, T follicular helper CD4+ T-cells have a memory that could potentially enhance the antibody-neutralising response against SARS-CoV-2. The memory of CD4+ and CD8+ T-cells might also enhance direct antiviral immunity in the lungs and nasopharynx early after exposure, consistent with previous knowledge that CD4+ T-cells in the lungs have antiviral activity against the associated SARS-CoV^85^ and that memory CD8+ T-cells protect against viral infections.

A recent study that used samples collected before the pandemic in Africa, Europe, and South and North America showed the occurrence of pre-existing humoral immunity against the spike (S) and nucleocapsid (N) proteins of SARS‑CoV‑2. Although antibodies specific to the SARS-CoV-2 S and N proteins were not common in all populations, the prevalence of N-specific antibodies was higher in the two African study locations, Gabon and Senegal, than in Europe and South and North America.^86^ In separate studies conducted with samples from Gabon (Central Africa), Nigeria, Ghana, Benin (West Africa), Tanzania (East Africa), and Zambia (Southern Africa), the pre-existing immunity was high against the SARS-CoV-2 N and, to a lower extent, S proteins,^87,88,89,90^ which suggests that high SARS-CoV-2 pre-existing immunity is widespread in Africa. In addition, previous studies conducted in France, England, and the United States have also reported low levels of pre-existing SARS-CoV-2 S protein cross-reactive antibodies in uninfected individuals.^91,92,93^

Although the reason for the underlying SARS-CoV-2 high seropositivity in African populations has not been established, some have postulated that the immune system could be stimulated to develop cross-reactive antibodies against SARS-CoV-2 as a result of infections with the common human coronaviruses^87,88^ due to the wide distribution of the common human coronaviruses throughout the globe.^14^ In this regard, samples collected from Africa, Europe, and South Africa were used in a recent serological study to demonstrate the existence of antibodies that cross-react against the N protein of common human coronaviruses, but their presence did not show any correlation with the SARS-CoV-2 N protein cross-reactive antibodies.^88^ This suggests the possibility that the high cross-reactive immunity reported by the African population is being driven by other main contributors besides the common human coronaviruses. High seropositivity against SARS-CoV-2 N in samples collected before the pandemic could also be an indication of the circulation of other coronaviruses that have not yet been recognised. The limited number of cases and mortality due to SARS-CoV-2 infection in Africa may be attributed to a high cross-reactive immunity background as observed in sera from African populations. Large studies are needed to measure and demonstrate the correlation between pre-existing immunity, prospective infection and disease severity as a way to define the possible function of pre-existing T-cell memory in SARS-CoV-2 infection.

### Innate immunity-mediated trained immunity and immune tolerance

In addition to adaptive immunity-mediated cross-immunity, trained immunity and immune tolerance mediated by the innate immune system could also considerably modify the clinical spectrum and mortality of COVID-19 infection. Trained immunity is the memory characteristic in the innate immune system of vertebrates that elicits an increased reaction to secondary infections or sterile activation of inflammation.^65,94^ Besides trained immunity, the cells of the innate immune system could also reach a status of immune tolerance or hypo-responsiveness, wherein there is a decrease in the production of pro-inflammatory mediators when these cells encounter secondary heterologous stimuli.^70,71,72^ For example, epidemiological data, as well as in vivo and in vitro studies with BCG vaccines conducted among humans and in murine-derived peripheral blood mononuclear cells subjected to immune-stimulatory agents such as lipopolysaccharide, β-glucan, and flagellin, provided evidence of either increased or decreased inflammatory reaction to secondary stimuli that corroborates the concept of trained immunity and immune tolerance.^70,71,72^ In addition, animal studies have shown that immunisation with the BCG vaccine can protect against subsequent infections by *Candida albicans* and *M. tuberculosis.*^95,96^ Immunisation with the BCG vaccine can also protect against a controlled version of human yellow fever infection^97^ or malaria^98^ by augmenting the pro-inflammatory activity of monocytes. It is reasonable to argue that the non-specific nature of this type of cross-protection is not consistent with mediation by adaptive immunity, but it demonstrates that the host defence can develop innate-like immune adaptation mechanisms.

Several countries have used the BCG vaccine to protect against tuberculosis caused by *M. tuberculosis* by exposing individuals to a live, attenuated strain of *Mycobacterium bovis*. Apart from the partial and possibly variable immunity the BCG vaccine offers against tuberculosis, it also protects in an off-target manner against other non‑tuberculosis infectious diseases.^99^ For example, BCG vaccination-induced trained immunity offers protection against infections with RNA and DNA viruses such as herpes and influenza, reduces yellow fever viraemia, and protects against respiratory tract infections.^97,100,101,102,103^ Data from a randomised, double-blind, clinical trial conducted recently showed that there was a reduction in the incidence of respiratory tract infections in BCG-vaccinated patients 65 years and older.^104^ This protection against a variety of pathogens is achieved through trained immunity.^97^

Most countries with a high incidence of tuberculosis have implemented a comprehensive BCG vaccination policy that encourages children to get the BCG vaccine in early childhood. The protection offered by the BCG vaccine may persist for up to 30–40 years after inoculation as described in a study in Norway,^105^ or up to 50–60 years as shown in a BCG vaccine trial among American Indians and Alaska Natives.^106^ However, data from these and some other studies have also shown that BCG vaccination-induced protection could also wane over time.^107^ Apart from its reported prospect to enhance innate immunity, preliminary epidemiological analyses have also shown a reduction in COVID-19 severity in countries that are implementing the policy of widespread BCG vaccination.^108^ These observations suggest that the BCG vaccine may protect against SARS-CoV-2 infection. Consequently, in 2020, BCG vaccination was proposed as a strategy to prevent infection and combat the COVID‑19 outbreak.^108^

Although BCG vaccination is linked to a reduction in the number of COVID-19 cases and mortality,^109,110,111^ many of the assessments do not consider likely confounding factors such as testing rate, socio-economic factors, and co-morbidities. One epidemiological study that corrected for confounding variables, particularly the testing rate, did not find evidence that the overall BCG vaccination policy correlated with the spread of SARS-CoV-2 and its associated mortality.^112^ Available information as of 2020 on the BCG Atlas (www.bcgatlas.org) showed that all African countries except South Sudan have an existing universal BCG vaccination policy. If it is proven that BCG vaccination does indeed offer protection against SARS-CoV-2, the relatively low number of cases and deaths could be attributed to the long-standing universal BCG vaccination policy in African countries rather than under-reporting or diagnostic limitations.

The determinant of whether the innate immune system exhibits either trained immunity or immune tolerance is not known but is thought to be related to the dose and duration of the primary trigger.^72,113^ Trained immunity and immune tolerance have been demonstrated in studies that focused on circulating mononuclear cells with a short lifespan, as well as progenitor stem cells of the bone marrow, epithelial cells, and resident tissue macrophages with long lifespans.^70,71^ This may imply that the host’s innate immune response to different pathogens could last longer.

Trained immunity and immune tolerance may have significant implications for COVID-19 infection. In sub-Saharan Africa, vast portions of the population live under unfavourable conditions such as crowded and unhygienic environments, leading to recurrent infections with bacteria, viruses, and eukaryotic parasites, and thus making it possible to attain immune tolerance against a novel pathogen like SARS-CoV-2. Immune tolerance could prevent the innate immune system from reaching a hyper-inflammatory state or a ‘cytokine storm’, which is generally associated with the ultimate fatality of COVID-19 disease.

People living in developed countries with better sanitary conditions are less exposed to multiple pathogens during their lifetime and thus less likely to attain immune tolerance. This may be partially responsible for the clinical spectrum and the heightened vulnerability to novel pathogens like SARS-CoV-2 observed in such populations.

## Projections into the future of COVID-19 and disease processes

Against the background of increasing population immunity due to vaccination and high rates of natural infection, many new variants of SARS-CoV-2 have emerged and have been designated by the WHO as either variants of concern or variants of interest.^114^ These variants pose a greater risk to public health because they may be more transmissible or produce more severe disease, escape immune response, cause diagnostic or treatment failure, or reduce the efficacy of vaccines.^114,115^ The five main variants of concern are: B.1.1.7 (Alpha), first described in the United Kingdom; B.1.351 (Beta), first reported in South Africa; P.1 (Gamma), originated from Brazil; B.1.617.2 (Delta), first described in India; and B.1.1.529 (Omicron), first detected in South Africa. The Alpha, Beta, and Gamma variants share the N501Y mutation, which likely makes them more transmissible. The E484K and K417N mutations displayed by both the Beta and Gamma variants decrease the binding of neutralising antibodies, thus causing partial immune escape, favouring reinfections, and reducing the in vitro efficacy of some antibody therapies or vaccines.^116,117^ The frequent emergence of these variants globally challenges the use of herd immunity as an approach to managing the pandemic and has been responsible for new waves of the pandemic and thousands of deaths globally.^114^ The Alpha variant was quickly replaced by the highly contagious Delta variant, which was soon outcompeted by the Omicron variant to become the dominant circulating variant.

The Omicron variant is more highly transmissible than the other variants, being able to infect 3–6 times more people than the Delta variant, including individuals immune to the other variants.^117,118^ The Omicron variant has been detected in 77 countries as of December 2021.^119,120^ Although several aspects of the behaviour of the Omicron variant and how it will impact the course of the pandemic have not been well defined, current data indicate that immunisation with existing vaccines could reduce the risk of serious illness, hospitalisation, and death.^119,121^ However, in vitro studies indicate that the neutralisation efficacy of vaccine sera against Omicron reduces greatly compared to the previously circulating Delta variant.^122^ If more data confirm that the Omicron variant indeed produces milder forms of the disease, it may suggest a trend that the pandemic is on its way out but the world may have to come to terms with living with the virus. It may require occasional vaccination like in the case of the flu virus to prevent infection. The background of previous exposure to multiple pathogens in the African population may provide a further advantage in conferring protection against the severe form of the disease.

In Africa, the prospect that new diseases may emerge is high due to the existing burden of infectious diseases and conducive environmental conditions such as high population growth, rapid economic development, increased exploitation of previously untapped natural resources, and increasing interactions between humans and wildlife. A prompt and coordinated global health response would be required to quickly control outbreaks either at the local or regional level as exemplified by the global response to the 2014 Ebola epidemic.^61,122^ While the emergence of new diseases may not have a major repercussion on African population dynamics, it may have serious economic and social consequences. When these diseases emerge, we can draw on scientific advances and previous experience, especially with HIV/AIDS and Ebola, to quickly contain them. Africa might be able to withstand some of these emerging diseases owing to enhanced immunity due to prior exposure to numerous pathogens and extensive vaccination programmes within the continent.

## Conclusion

This review has shown that pre-existing immunity against SARS-CoV-2 is widespread in Africa and higher than in Western countries. This immunity may be related to previous exposure to endemic coronaviruses or other multiple pathogens and could have been responsible for the reduced disease transmission and the limited number of cases and mortality recorded in African countries. The relatively low number of cases and deaths in Africa could be attributed to the activation of innate immune-mediated trained immunity or immune tolerance due to frequent exposure to multiple pathogens and the impact of long-standing universal BCG vaccination policies in many African countries rather than under-reporting or diagnostic limitations.

## References

[1] World Health Organization (WHO). Novel coronavirus – China [homepage on the Internet]. 2020 [cited 2021 Sept 21]. Available from: https://www.who.int/emergencies/disease-outbreak-news/item/2020-DON233

[2] Gorbalenya AE, Baker SC, Baric RS, et al. The species severe acute respiratory syndrome-related coronavirus: Classifying 2019-nCoV and naming it SARS-CoV-2. Nat Microbiol. 2020;5(4):536–544. 10.1038/s41564-020-0695-z 32123347 PMC7095448

[3] World Health Organization (WHO). WHO coronavirus (COVID-19) dashboard [homepage on the Internet]. 2020 [cited 2021 Sept 21]. Available from: https://covid19.who.int

[4] Chen N, Zhou M, Dong X, et al. Epidemiological and clinical characteristics of 99 cases of 2019 novel coronavirus pneumonia in Wuhan, China: A descriptive study. Lancet. 2020;395(10223):507–513. 10.1016/S0140-6736(20)30211-7 32007143 PMC7135076

[5] Huang C, Wang Y, Li X, et al. Clinical features of patients infected with 2019 novel coronavirus in Wuhan, China. Lancet. 2020;395(10223):497–506. 10.1016/S0140-6736(20)30183-5 31986264 PMC7159299

[6] Hu B, Guo H, Zhou P, Shi Z-L. Characteristics of SARS-CoV-2 and COVID-19. Nat Rev Microbiol. 2020;19:141–154. 10.1038/s41579-020-00459-7 33024307 PMC7537588

[7] Chan JF-W, Yuan S, Kok K-H, et al. A familial cluster of pneumonia associated with the 2019 novel coronavirus indicating person-to-person transmission: A study of a family cluster. Lancet. 2020;395(10223):514–523. 10.1016/S0140-6736(20)30154-9 31986261 PMC7159286

[8] Nishiura H, Kobayashi T, Miyama T, et al. Estimation of the asymptomatic ratio of novel coronavirus infections (COVID-19). Int J Infect Dis. 2020;94:154–155. 10.1016/j.ijid.2020.03.020 32179137 PMC7270890

[9] Mizumoto K, Kagaya K, Zarebski A, Chowell G. Estimating the asymptomatic proportion of coronavirus disease 2019 (COVID-19) cases on board the Diamond Princess cruise ship, Yokohama, Japan, 2020. Euro Surveill Bull Eur Sur Mal Transm Eur Commun Dis Bull. 2020;25(10):2000180. 10.2807/1560-7917.ES.2020.25.10.2000180 PMC7078829 32183930

[10] Wang F, Nie J, Wang H, et al. Characteristics of peripheral lymphocyte subset alteration in COVID-19 Pneumonia. J Infect Dis. 2020;221(11):1762–1769. 10.1093/infdis/jiaa150 32227123 PMC7184346

[11] Bai Y, Yao L, Wei T, et al. Presumed asymptomatic carrier transmission of COVID-19. JAMA. 2020;323(14):1406–1407. 10.1001/jama.2020.2565 32083643 PMC7042844

[12] Weiss SR, Leibowitz JL. Coronavirus pathogenesis. Adv Virus Res. 2011; 81:85–164. 10.1016/B978-0-12-385885-6.00009-2 22094080 PMC7149603

[13] Knipe DM, Howley PM. Fields virology: Editors-in-chief, David M. Knipe, Peter M. Howley. Philadelphia, PA: Wolters Kluwer/Lippincott Williams & Wilkins Health; 2013.

[14] Su S, Wong G, Shi W, et al. Epidemiology, genetic recombination, and pathogenesis of coronaviruses. Trends Microbiol. 2016;24(6):490–502. 10.1016/j.tim.2016.03.003 27012512 PMC7125511

[15] Zhong NS, Zheng BJ, Li YM, et al. Epidemiology and cause of severe acute respiratory syndrome (SARS) in Guangdong, People’s Republic of China, in February, 2003. Lancet. 2003;362(9393):1353–1358. 10.1016/S0140-6736(03)14630-2 14585636 PMC7112415

[16] Ksiazek TG, Erdman D, Goldsmith CS, et al. A novel coronavirus associated with severe acute respiratory syndrome. N Engl J Med. 2003;348(20):1953–1366. 10.1056/NEJMoa030781 12690092

[17] Drosten C, Günther S, Preiser W, et al. Identification of a novel coronavirus in patients with severe acute respiratory syndrome. N Engl J Med. 2003;348(20):1967–1976. 10.1056/NEJMoa030747 12690091

[18] Zaki AM, Van Boheemen S, Bestebroer TM, Osterhaus ADME, Fouchier RAM. Isolation of a novel coronavirus from a man with pneumonia in Saudi Arabia. N Engl J Med. 2012;367(19):1814–1820. 10.1056/NEJMoa1211721 23075143

[19] Cui J, Li F, Shi Z-L. Origin and evolution of pathogenic coronaviruses. Nat Rev Microbiol. 2019;17(3):181–192. 10.1038/s41579-018-0118-9 30531947 PMC7097006

[20] Wong G, Liu W, Liu Y, Zhou B, Bi Y, Gao GF. MERS, SARS, and Ebola: The role of super-spreaders in infectious disease. Cell Host Microbe. 2015;18(4):398–401. 10.1016/j.chom.2015.09.013 26468744 PMC7128246

[21] Dan JM, Mateus J, Kato Y, et al. Immunological memory to SARS-CoV-2 assessed for up to 8 months after infection. Science. 2021;371(6529): eabf4063. 10.1126/science.abf4063 33408181 PMC7919858

[22] Grifoni A, Weiskopf D, Ramirez SI, et al. Targets of T cell responses to SARS-CoV-2 coronavirus in humans with COVID-19 disease and unexposed individuals. Cell. 2020;181(7):1489–1501.e15. 10.1016/j.cell.2020.05.015 32473127 PMC7237901

[23] Weiskopf D, Schmitz KS, Raadsen MP, et al. Phenotype and kinetics of SARS-CoV-2-specific T cells in COVID-19 patients with acute respiratory distress syndrome. Sci Immunol. 2020;5(48): eabd2071. 10.1126/sciimmunol.abd2071 32591408 PMC7319493

[24] Braun J, Loyal L, Frentsch M, et al. SARS-CoV-2-reactive T cells in healthy donors and patients with COVID-19. Nature. 2020;587(7833):270–274. 10.1038/s41586-020-2598-9 32726801

[25] Liu W, Liu L, Kou G, et al. Evaluation of Nucleocapsid and spike protein-based enzyme-linked immunosorbent assays for detecting antibodies against SARS-CoV-2. J Clin Microbiol. 2020;58(6): e00461-20. 10.1128/JCM.00461-20 32229605 PMC7269413

[26] Zhao J, Yuan Q, Wang H, et al. Antibody responses to SARS-CoV-2 in patients of novel coronavirus disease 2019. Clin Infect Dis. 2020;71(16):2027–2034. 10.1101/2020.03.02.20030189 32221519 PMC7184337

[27] Okba NMA, Müller MA, Li W, et al. Severe acute respiratory syndrome coronavirus 2-specific antibody responses in coronavirus disease patients. Emerg Infect Dis. 2020;26(7):1478–1488. 10.3201/eid2607.200841 32267220 PMC7323511

[28] Long Q-X, Liu B-Z, Deng H-J, et al. Antibody responses to SARS-CoV-2 in patients with COVID-19. Nat Med. 2020;26(6):845–848. 10.1038/s41591-020-0897-1 32350462

[29] Wölfel R, Corman VM, Guggemos W, et al. Virological assessment of hospitalized patients with COVID-2019. Nature. 2020;581(7809):465–469. 10.1038/s41586-020-2196-x 32235945

[30] Orenstein WA, Ahmed R. Simply put: Vaccination saves lives. Proc Natl Acad Sci U S A. 2017;114(16):4031–4033. 10.1073/pnas.1704507114 28396427 PMC5402432

[31] Plotkin S, Orenstein WA, Offit P, Edwards KM. Plotkin’s vaccines. 7th ed. [homepage on the Internet]. 2017 [cited 2021 Sept 22]. Available from: https://www.elsevier.com/books/T/A/9780323357616

[32] Piot P, Larson HJ, O’Brien KL, et al. Immunization: Vital progress, unfinished agenda. Nature. 2019;575(7781):119–129. 10.1038/s41586-019-1656-7 31695203

[33] Kellam P, Barclay W. The dynamics of humoral immune responses following SARS-CoV-2 infection and the potential for reinfection. J Gen Virol. 2020;101(8):791–797. 10.1099/jgv.0.001439 32430094 PMC7641391

[34] Wu L-P, Wang N-C, Chang Y-H, et al. Duration of antibody responses after severe acute respiratory syndrome. Emerg Infect Dis. 2007;13(10):1562–1564. 10.3201/eid1310.070576 18258008 PMC2851497

[35] Xiao AT, Gao C, Zhang S. Profile of specific antibodies to SARS-CoV-2: The first report. J Infect. 2020;81(1):147–178. 10.1016/j.jinf.2020.03.012 PMC7118534 32209385

[36] Zeng H, Xu C, Fan J, et al. Antibodies in infants born to mothers with COVID-19 pneumonia. JAMA. 2020;323(18):1848–1849. 10.1001/jama.2020.4861 32215589 PMC7099444

[37] Africa CDC. Outbreak brief 86: Coronavirus disease 2019 (COVID-19) pandemic [homepage on the Internet]. Africa CDC; 2021 [cited 2022 Sept 27]. Available from: https://africacdc.org/download/outbreak-brief-86-coronavirus-disease-2019-covid-19-pandemic/

[38] Embrett M, Sim SM, Caldwell HAT, et al. Barriers to and strategies to address COVID-19 testing hesitancy: A rapid scoping review. BMC Public Health. 2022;22(1):750. 10.1186/s12889-022-13127-7 35422031 PMC9008387

[39] Kaseje N. Why sub-Saharan Africa needs a unique response to COVID-19 [homepage on the Internet]. World Economic Forum; 2020 [cited 2021 Nov 24]. Available from: https://www.weforum.org/agenda/2020/03/why-sub-saharan-africa-needs-a-unique-response-to-covid-19/

[40] Centers for Disease Control and Prevention (CDC). COVID-19 and your health [homepage on the Internet]. Centers for Disease Control and Prevention; 2020 [cited 2021 Nov 24]. Available from: https://www.cdc.gov/coronavirus/2019-ncov/need-extra-precautions/people-with-medical-conditions.html

[41] Kaneda T, Ashford LS. Sub-Saharan Africa’s demographic and health characteristics will influence the course of the COVID-19 pandemic – Population reference bureau [homepage on the Internet]. 2020 [cited 2020 Aug 14]. Available from: https://www.prb.org/sub-saharan-africas-demographic-and-health-characteristics-will-influence-the-course-of-the-covid-19-pandemic/

[42] Gona PN, Gona CM, Ballout S, et al. Burden and changes in HIV/AIDS morbidity and mortality in Southern Africa development community countries, 1990–2017. BMC Public Health. 2020;20(1):867. 10.1186/s12889-020-08988-9 32503604 PMC7274054

[43] Dye C, Harries AD, Maher D, Hosseini SM, Nkhoma W, Salaniponi FM. Tuberculosis. In: Jamison DT, Feachem RG, Makgoba MW, et al., editors. Disease and mortality in sub-Saharan Africa [homepage on the Internet]. 2nd ed. Washington, DC: World Bank; 2006 [cited 2021 Nov 23]. Available from: http://www.ncbi.nlm.nih.gov/books/NBK2285/

[44] Young F, Critchley JA, Johnstone LK, Unwin NC. A review of co-morbidity between infectious and chronic disease in sub Saharan Africa: TB and diabetes mellitus, HIV and metabolic syndrome, and the impact of globalization. Glob Health. 2009;5:9. 10.1186/1744-8603-5-9 PMC2753337 19751503

[45] Corburn J, Hildebrand C. Slum sanitation and the social determinants of women’s health in Nairobi, Kenya. J Environ Public Health. 2015;2015: e209505. 10.1155/2015/209505 PMC4427764 26060499

[46] United Nations Human Settlements Programme (UN-Habitat). COVID-19 in African cities: Impacts, Responses and Policies 2020. Available from: https://unhabitat.org/covid-19-in-africa-cities-impacts-responses-and-policies

[47] UNICEF. Water, sanitation and hygiene [home on the Internet]. 2021 [cited 2021 Nov 23]. Available from: https://www.unicef.org/wca/what-we-do/wash

[48] Boutayeb A. The impact of infectious diseases on the development of Africa. In: Preedy VR, Watson RR, editors. Handbook of disease burdens and quality of life measures. New York, NY: Springer; 2010, pp. 1171–1188.

[49] World Health Organization (WHO). Global health estimates: Leading causes of DALYs [Internet]. 2020 [cited 2022 Oct 31]. Available from: https://www.who.int/data/gho/data/themes/mortality-and-global-health-estimates/global-health-estimates-leading-causes-of-dalys

[50] World Health Organization (WHO). World malaria report 2020 [homepage on the Internet]. 2020 [cited 2021 Sept 23]. Available from: https://www.who.int/teams/global-malaria-programme/reports/world-malaria-report-2020

[51] Picker LJ, Watkins DI. HIV pathogenesis: The first cut is the deepest. Nat Immunol. 2005;6(5):430–432. 10.1038/ni0505-430 15843796

[52] Naif HM. Pathogenesis of HIV infection. Infect Dis Rep. 2013;5(suppl. 1):e6. 10.4081/idr.2013.s1.e6 PMC3892619 24470970

[53] UNAIDS. Global HIV & AIDS statistics – Fact sheet [home on the Internet]. 2020 [cited 2021 Sept 23]. Available from: https://www.unaids.org/en/resources/fact-sheet

[54] World Health Organization (WHO). Tuberculosis [homepage on the Internet]. 2018 [cited 2021 Nov 07]. Available from: https://www.who.int/news-room/q-a-detail/tuberculosis

[55] Toth A, Fackelmann J, Pigott W, Tolomeo O. Tuberculosis prevention and treatment. Can Nurse. 2004;100(9):27–30. 15623010

[56] World Health Organization (WHO). Global tuberculosis report 2020 [homepage on the Internet]. 2020 [cited 2021 Sept 23]. Available from: https://www.who.int/publications/i/item/9789240013131

[57] World Health Organization (WHO). Ebola virus disease [home on the Internet]. 2021 [cited 2021 Sept 23]. Available from: https://www.who.int/news-room/fact-sheets/detail/ebola-virus-disease

[58] Baseler L, Chertow DS, Johnson KM, Feldmann H, Morens DM. The pathogenesis of Ebola virus disease. Ann Rev Pathol. 2017;12:387–418. 10.1146/annurev-pathol-052016-100506 27959626

[59] World Health Organization (WHO). Ebola haemorrhagic fever in Zaire, 1976. Bull World Health Organ. 1978;56(2):271–293. 307456 PMC2395567

[60] Rouquet P, Froment J-M, Bermejo M, et al. Wild animal mortality monitoring and human Ebola outbreaks, Gabon and Republic of Congo, 2001–2003. Emerg Infect Dis. 2005;11(2):283–290. 10.3201/eid1102.040533 15752448 PMC3320460

[61] CDC. 2014–2016 Ebola Outbreak in West Africa | History | Ebola (Ebola Virus Disease) | CDC [home on the Internet]. 2016 [cited 2021 Sept 23]. Available from: https://www.cdc.gov/vhf/ebola/history/2014-2016-outbreak/index.html

[62] Coltart CEM, Lindsey B, Ghinai I, Johnson AM, Heymann DL. The Ebola outbreak, 2013–2016: Old lessons for new epidemics. Philos Trans R Soc B Biol Sci. 2017;372(1721):20160297. 10.1098/rstb.2016.0297 PMC5394636 28396469

[63] Rojek A, Horby P, Dunning J. Insights from clinical research completed during the West Africa Ebola virus disease epidemic. Lancet Infect Dis. 2017;17(9): e280–e292. 10.1016/S1473-3099(17)30234-7 28461209 PMC5856335

[64] Iwasaki A, Medzhitov R. Control of adaptive immunity by the innate immune system. Nat Immunol. 2015;16(4):343–353. 10.1038/ni.3123 25789684 PMC4507498

[65] Schenten D, Medzhitov R. The control of adaptive immune responses by the innate immune system. Adv Immunol. 2011;109:87–124. 10.1016/B978-0-12-387664-5.00003-0 21569913

[66] Dianzani F, Baron S. Nonspecific defenses. In: Baron S, editor. Medical microbiology [homepage on the Internet]. 4th ed. Galveston, TX: University of Texas Medical Branch at Galveston; 1996 [cited 2021 Nov 21]. Available from: http://www.ncbi.nlm.nih.gov/books/NBK8348/21413325

[67] Klimpel GR. Immune defenses. In: Baron S, editor. Medical microbiology [homepage on the Internet]. 4th ed. Galveston, TX: University of Texas Medical Branch at Galveston; 1996 [cited 2021 Nov 21]. Available from: http://www.ncbi.nlm.nih.gov/books/NBK8423/21413332

[68] Netea MG, Domínguez-Andrés J, Barreiro LB, et al. Defining trained immunity and its role in health and disease. Nat Rev Immunol. 2020;20(6):375–388. 10.1038/s41577-020-0285-6 32132681 PMC7186935

[69] Netea MG, Quintin J, Van der Meer JWM. Trained immunity: A memory for innate host defense. Cell Host Microbe. 2011;9(5):355–361. 10.1016/j.chom.2011.04.006 21575907

[70] Divangahi M, Aaby P, Khader SA, et al. Trained immunity, tolerance, priming and differentiation: Distinct immunological processes. Nat Immunol. 2021;22(1):2–6. 10.1038/s41590-020-00845-6 33293712 PMC8020292

[71] Peignier A, Parker D. Trained immunity and host-pathogen interactions. Cell Microbiol. 2020;22(12): e13261. 10.1111/cmi.13261 32902895 PMC7655595

[72] Ifrim DC, Quintin J, Joosten LAB, et al. Trained immunity or tolerance: Opposing functional programs induced in human monocytes after engagement of various pattern recognition receptors. Clin Vaccine Immunol. 2014;21(4):534–545. 10.1128/CVI.00688-13 24521784 PMC3993125

[73] Kleinnijenhuis J, Quintin J, Preijers F, et al. Bacille Calmette-Guerin induces NOD2-dependent nonspecific protection from reinfection via epigenetic reprogramming of monocytes. Proc Natl Acad Sci U S A. 2012;109(43):17537–17542. 10.1073/pnas.1202870109 22988082 PMC3491454

[74] Lerm M, Netea MG. Trained immunity: A new avenue for tuberculosis vaccine development. J Intern Med. 2016;279(4):337–346. 10.1111/joim.12449 26602369

[75] Sánchez-Ramón S, Conejero L, Netea MG, Sancho D, Palomares Ó, Subiza JL. Trained immunity-based vaccines: A new paradigm for the development of broad-spectrum anti-infectious formulations. Front Immunol. 2018;9:2936. 10.3389/fimmu.2018.02936 30619296 PMC6304371

[76] Arts RJW, Joosten LAB, Netea MG. The potential role of trained immunity in autoimmune and auto inflammatory disorders. Front Immunol. 2018;9:298. 10.3389/fimmu.2018.00298 29515591 PMC5826224

[77] Yuki K, Fujiogi M, Koutsogiannaki S. COVID-19 pathophysiology: A review. Clin Immunol. 2020;215:108427. 10.1016/j.clim.2020.108427 32325252 PMC7169933

[78] Chakrabarti SS, Kaur U, Singh A, et al. Of cross-immunity, herd immunity and country-specific plans: Experiences from COVID-19 in India. Aging Dis. 2020;11(6):1339. 10.14336/AD.2020.1104 33269091 PMC7673860

[79] Gorse GJ, Patel GB, Vitale JN, O’Connor TZ. Prevalence of antibodies to four human coronaviruses is lower in nasal secretions than in serum. Clin Vaccine Immunol. 2010;17(12):1875–1880. 10.1128/CVI.00278-10 20943876 PMC3008199

[80] Lipsitch M, Grad YH, Sette A, Crotty S. Cross-reactive memory T cells and herd immunity to SARS-CoV-2. Nat Rev Immunol. 2020;20(11):709–713. 10.1038/s41577-020-00460-4 33024281 PMC7537578

[81] Le Bert N, Tan AT, Kunasegaran K, et al. SARS-CoV-2-specific T cell immunity in cases of COVID-19 and SARS, and uninfected controls. Nature. 2020;584(7821):457–462. 10.1038/s41586-020-2550-z 32668444

[82] Stefano GB, Kream RM. Convalescent memory T cell immunity in individuals with mild or asymptomatic SARS-CoV-2 infection may result from an evolutionarily adapted immune response to coronavirus and the “common cold.” Int Med J Exp Clin Res. 2020;26: e929789. 10.12659/MSM.929789 PMC7706138 33239605

[83] Woldemeskel BA, Kwaa AK, Garliss CC, Laeyendecker O, Ray SC, Blankson JN. Healthy donor T cell responses to common cold coronaviruses and SARS-CoV-2. J Clin Invest. 2020;130(12):6631–6638. 10.12659/MSM.929789 32966269 PMC7685719

[84] Chakrabarti S, Chakrabarti SS, Kaur U, Agrawal BK, Ganguly U, Jin K. Cross-immunity and trained immunity in explaining variable COVID-19 mortality – Guidance for future pandemics. J Med Virol. 2021;93(7):4094–4096. 10.1002/jmv.26958 33755226 PMC8251060

[85] Zhao J, Zhao J, Mangalam AK, et al. Airway memory CD4(+) T cells mediate protective immunity against emerging respiratory coronaviruses. Immunity. 2016;44(6):1379–1391. 10.1016/j.immuni.2016.05.006 27287409 PMC4917442

[86] Pedersen J, Koumakpayi IH, Babuadze G, et al. Cross-reactive immunity against SARS-CoV-2 N protein in Central and West Africa precedes the COVID-19 pandemic. Sci Rep.;12(1):12962. 10.1038/s41598-022-17241-9 PMC9333058 35902675

[87] Tso FY, Lidenge SJ, Peña PB, et al. High prevalence of pre-existing serological cross-reactivity against severe acute respiratory syndrome coronavirus-2 (SARS-CoV-2) in sub-Saharan Africa. Int J Infect Dis. 2021;102:577–583. 10.1016/j.ijid.2020.10.104 33176202 PMC7648883

[88] Emmerich P, Murawski C, Ehmen C, et al. Limited specificity of commercially available SARS-CoV-2 IgG ELISAs in serum samples of African origin. Trop Med Int Health. 2021;26(6):621–631. 10.1111/tmi.13569 33666297 PMC8014856

[89] Mveang Nzoghe A, Essone PN, Leboueny M, et al. Evidence and implications of pre-existing humoral cross-reactive immunity to SARS-CoV-2. Immun Inflamm Dis. 2021;9(1):128–133. 10.1002/iid3.367 33320447 PMC7860591

[90] Yadouleton A, Sander AL, Moreira-Soto A, et al. Limited specificity of serologic tests for SARS-CoV-2 antibody detection, Benin. Emerg Infect Dis. 2021;27(1). 10.3201/eid2701.203281 PMC7774555 33261717

[91] Grzelak L, Temmam S, Planchais C, et al. A comparison of four serological assays for detecting anti-SARS-CoV-2 antibodies in human serum samples from different populations. Sci Transl Med. 2020;12(559): eabc3103. 10.1126/scitranslmed.abc3103 32817357 PMC7665313

[92] Ng KW, Faulkner N, Cornish GH, et al. Preexisting and de novo humoral immunity to SARS-CoV-2 in humans. Science. 2020;370(6522):1339–1343. 10.1126/science.abe1107 33159009 PMC7857411

[93] Song G, He W-T., Callaghan S, et al. Cross-reactive serum and memory B-cell responses to spike protein in SARS-CoV-2 and endemic coronavirus infection. Nat Commun. 2021;12(1):2938. 10.1038/s41467-021-23074-3 34011939 PMC8134462

[94] Netea MG, Joosten LAB, Latz E, et al. Trained immunity: A program of innate immune memory in health and disease. Science. 2016;352(6284): aaf1098. 10.1126/science.aaf1098 27102489 PMC5087274

[95] Van’t Wout JW, Poell R, Van Furth R. The role of BCG/PPD-activated macrophages in resistance against systemic candidiasis in mice. Scand J Immunol. 1992;36(5):713–719. 10.1111/j.1365-3083.1992.tb03132.x 1439583

[96] Kaufmann E, Sanz J, Dunn JL, et al. BCG educates hematopoietic stem cells to generate protective innate immunity against tuberculosis. Cell. 2018;172(1–2):176–190.e19. 10.1016/j.cell.2017.12.031 29328912

[97] Arts RJW, Moorlag SJCFM, Novakovic B, et al. BCG vaccination protects against experimental viral infection in humans through the induction of cytokines associated with trained immunity. Cell Host Microbe. 2018;23(1):89–100.e5. 10.1016/j.chom.2017.12.010 29324233

[98] Walk J, De Bree LCJ, Graumans W, et al. Outcomes of controlled human malaria infection after BCG vaccination. Nat Commun. 2019;10(1):874. 10.1038/s41467-019-08659-3 30787276 PMC6382772

[99] Tanner R, Villarreal-Ramos B, Vordermeier HM, McShane H. The humoral immune response to BCG vaccination. Front Immunol. 2019;10:1317. 10.3389/fimmu.2019.01317 31244856 PMC6579862

[100] Moorlag SJCFM, Arts RJW, Van Crevel R, Netea MG. Non-specific effects of BCG vaccine on viral infections. Clin Microbiol Infect. 2019;25(12):1473–1478. 10.1016/j.cmi.2019.04.020 31055165

[101] Arnoldussen DL, Linehan M, Sheikh A. BCG vaccination and allergy: A systematic review and meta-analysis. J Allergy Clin Immunol. 2011;127(1):246–53, 253.e1–e21. 10.1016/j.jaci.2010.07.039 20933258

[102] Nakayama K, Monma M, Fukushima T, Ohrui T, Sasaki H. Tuberculin responses and risk of pneumonia in immobile elderly patients. Thorax. 2000;55(10):867–869. 10.1136/thorax.55.10.867 10992541 PMC1745620

[103] Ohrui T, Nakayama K, Fukushima T, Chiba H, Sasaki H. [Prevention of elderly pneumonia by pneumococcal, influenza and BCG vaccinations]. Nihon Ronen Igakkai Zasshi Jpn J Geriatr. 2005;42(1):34–36. 10.3143/geriatrics.42.34 15732353

[104] Giamarellos-Bourboulis EJ, Tsilika M, Moorlag S, et al. Activate: Randomized clinical trial of BCG vaccination against infection in the elderly. Cell. 2020;183(2):315–323.e9. 10.1016/j.cell.2020.08.051 32941801 PMC7462457

[105] Nguipdop-Djomo P, Heldal E, Rodrigues LC, Abubakar I, Mangtani P. Duration of BCG protection against tuberculosis and change in effectiveness with time since vaccination in Norway: A retrospective population-based cohort study. Lancet Infect Dis. 2016;16(2):219–226. 10.1016/S1473-3099(15)00400-4 26603173

[106] Aronson NE, Santosham M, Comstock GW, et al. Long-term efficacy of BCG vaccine in American Indians and Alaska Natives: A 60-year follow-up study. JAMA. 2004;291(17):2086–2091. 10.1001/jama.291.17.2086 15126436

[107] Menzies D. Interpretation of repeated tuberculin tests. Boosting, conversion, and reversion. Am J Respir Crit Care Med. 1999;159(1):15–21. 10.1164/ajrccm.159.1.9801120 9872812

[108] Miller A, Reandelar MJ, Fasciglione K, Roumenova V, Li Y, Otazu GH. Correlation between universal BCG vaccination policy and reduced morbidity and mortality for COVID-19: An epidemiological study [serial online]. 2020 [cited 2021 Nov 16]:20042937. Available from: https://www.medrxiv.org/content/10.1101/2020.03.24.20042937v1

[109] Levine DI. A shred of evidence that BCG vaccine may protect against COVID-19: Comparing cohorts in Spain and Italy [serial online]. 2020 [cited 2021 Nov 17]:20123539. Available from: https://www.medrxiv.org/content/10.1101/2020.06.05.20123539v1

[110] Curtis N, Sparrow A, Ghebreyesus TA, Netea MG. Considering BCG vaccination to reduce the impact of COVID-19. Lancet. 2020;395(10236):1545–1546. 10.1016/S0140-6736(20)31025-4 32359402 PMC7252177

[111] Brooks NA, Puri A, Garg S, et al. The association of coronavirus disease-19 mortality and prior bacille Calmette-Guerin vaccination: A robust ecological analysis using unsupervised machine learning. Sci Rep. 2021;11(1):774. 10.1038/s41598-020-80787-z 33436946 PMC7804196

[112] Hensel J, McAndrews KM, McGrail DJ, Dowlatshahi DP, LeBleu VS, Kalluri R. Protection against SARS-CoV-2 by BCG vaccination is not supported by epidemiological analyses. Sci Rep. 2020;10(1):18377. 10.1038/s41598-020-75491-x 33110184 PMC7591473

[113] Seeley JJ, Ghosh S. Molecular mechanisms of innate memory and tolerance to LPS. J Leukoc Biol. 2017;101(1):107–119. 10.1189/jlb.3MR0316-118RR 27780875

[114] World Health Organization (WHO). Tracking SARS-CoV-2 variants [homepage on the Internet]. 2021 [cited 2021 Dec 25]. Available from: https://www.who.int/en/activities/tracking-SARS-CoV-2-variants/

[115] Parums DV. Revised World Health Organization (WHO) terminology for variants of concern and variants of interest of SARS-CoV-2. Med Sci Monit. 2021;27: e933622. 10.12659/MSM.933622 34149046 PMC8230247

[116] Zahradník J, Marciano S, Shemesh M, et al. SARS-CoV-2 variant prediction and antiviral drug design are enabled by RBD *in vitro* evolution. Nat Microbiol. 2021;6:1188–1198. 10.1038/s41564-021-00954-4 34400835

[117] Ren SY, Wang WB, Gao RD, Zhou AM. Omicron variant (B.1.1.529) of SARS-CoV-2: Mutation, infectivity, transmission, and vaccine resistance. World J Clin Cases. 2022;10(1):1–11. 10.12998/wjcc.v10.i1.1 35071500 PMC8727245

[118] HKU Med. HKUMed finds Omicron SARS-CoV-2 can infect faster and better than Delta in human bronchus but with less severe infection in lung. Hong Kong: HKU Med.

[119] Callaway E. Omicron likely to weaken COVID vaccine protection. Nature. 2021;600:367–368. 10.1038/d41586-021-03672-3 34880488

[120] Callaway E, Ledford H. How bad is Omicron? Nature. 2021;600:197–199. 10.1038/d41586-021-03614-z 34857948

[121] Pfizer Inc. Pfizer and BioNTech provide update on Omicron variant [homepage on the Internet]. 2021 [cited 2021 Dec 09]. Available from: https://www.businesswire.com/news/home/20211208005542/en/Pfizer-and-BioNTech-Provide-Update-on-Omicron-Variant

[122] Wilhelm A, Widera M, Grikscheit K, et al. Reduced neutralization of SARS-CoV-2 Omicron variant by vaccine sera and monoclonal antibodies [homepage on the Internet]. 2021, p. 21267432 [cited 2021 Dec 09]. Available from: https://www.medrxiv.org/content/10.1101/2021.12.07.21267432v1

